# Delayed Diagnosis of Intracranial Trauma

**DOI:** 10.7759/cureus.47738

**Published:** 2023-10-26

**Authors:** Catherine A Marco, Tori Beth L Snoad, Collette Poisson, Avram Flamm

**Affiliations:** 1 Emergency Medicine, Penn State Health Milton S. Hershey Medical Center, Hershey, USA; 2 Emergency Medicine, WellSpan Health, York, USA

**Keywords:** diagnosis, brain trauma injury, trauma, emergency, head trauma

## Abstract

Introduction

Trauma is one of the leading causes of death and hospitalization in the United States. Head trauma often results in significant morbidity and mortality. This study was undertaken to identify reasons for delay in diagnosis of intracranial trauma.

Methods

This retrospective study analyzed patients with intracranial trauma between 2016 and 2022, in which there was a delay of two days or more from the date of injury to the date of diagnosis.

Results

Among 809 patients with head trauma, 140 subjects were identified with delayed diagnosis of intracranial trauma (17.3%). The most common diagnoses were subdural hemorrhage (N = 82; 56%) and intraparenchymal hemorrhage (N = 33; 24%). The most common reasons for delay in diagnosis included patient delay in seeking care (N = 111; 79%), and delayed diagnosis during inpatient hospitalization (N = 16; 11%) (Chi-Square <0.0001) (Table 2). Among inpatients with delayed diagnosis, confounding issues included alcohol intoxication (N = 4; 3%), other injuries (N = 9; 6%), and mental health issues (N = 2; 1%).

Conclusions

Among patients with delayed diagnosis of intracranial trauma, the majority of delays in diagnosis were due to patient delay in seeking care. Future directions may include improved public education regarding trauma and the importance of seeking timely medical care.

## Introduction

Trauma is one of the leading causes of death in the United States, accounting for over 700,000 hospitalizations in 2018 [1,2]. Head trauma is a common subset of trauma. Over 1.7 million traumatic brain injuries (TBI) occur annually [3]. Mechanisms of injury include falls, motor vehicle crashes, assaults, and sports-related injuries [4]. Head trauma, even mild, may be associated with significant or long-term effects, including fatigue, sleep disturbances, headache, and memory impairment [5,6]. Undiagnosed head trauma is associated with concussion-related symptoms [7].

Several studies have reported factors associated with occult trauma. A study by Parreira et al. found that occult intraabdominal trauma was associated with motorcycle accidents, rib fractures, run-over pedestrians, and abnormal neurologic examination [8]. Several studies have found that predictors of intracranial injury included physical findings of head trauma and a history of loss of consciousness [9,10].

This study was undertaken to identify the incidence of delayed diagnosis of intracranial trauma and the reasons for the delay in diagnosis.

## Materials and methods

This retrospective chart review was approved by the Penn State University Institutional Review Board as Exempt Research (approval number: 00021381). Data were extracted from the Pennsylvania Trauma Systems Foundation Data Base Collection System. Eligible subjects included trauma patients aged 18 years and older, with intracranial trauma, who presented to Penn State Health Milton S. Hershey Medical Center, Hershey, Pennsylvania, United States, between January 2016 and October 2022 and had a delay in diagnosis of intracranial trauma of 2-14 days following the date of the trauma. Eligible subjects were identified with a database search of patients with the date of injury two or more days prior to the day of diagnosis. Medical records were reviewed to extract data, including age, sex, race, ethnicity, date of injury, date of diagnosis, mechanism of injury, diagnosis, days of hospitalization, and outcome. Medical records were reviewed to characterize the reason for the delay in diagnosis. Reasons were coded as one or more of the following: Patient did not seek care, Diagnosis not made at initial ED visit, Diagnosis not made at other outpatient facilities, Diagnosis not made initially during inpatient hospitalization, Alcohol intoxication, Other injuries, or Mental health issue. Data entries were verified for accuracy by random chart quality review.

Data analysis

Descriptive statistics were generated including means, medians, standard deviations, and 95% confidence intervals. The statistical analysis was performed using SAS software, version 9.4 (2016; SAS Institute Inc., Cary, North Carolina, United States).

## Results

Among 809 patients with head trauma, 140 subjects were identified with delayed diagnosis of intracranial trauma (17.3%). The mean age was 66 years and the majority were male (N = 88; 63%). The most common diagnoses were subdural hemorrhage (N = 82; 56%) and intraparenchymal hemorrhage (N = 33; 24%) (Table 1). 

**Table 1:** Diagnoses of 140 Subjects with Delayed Diagnosis of Intracranial Trauma* *Totals >140, due to some subjects with multiple diagnoses

Diagnosis	N	%
Subdural hemorrhage	82	59
Intraparenchymal hemorrhage	33	24
Subarachnoid hemorrhage	22	16
Traumatic brain Injury	21	15
Concussion	19	14
Skull fracture	12	9
Epidural hemorrhage	9	6

The most common reasons for delay in diagnosis included patient delay in seeking care (N = 111; 79%), and delayed diagnosis during inpatient hospitalization (N = 16; 11%). Among inpatients with delayed diagnosis, confounding issues included alcohol intoxication (N = 4; 3%), other injuries (N = 9; 6%), and mental health issues (N = 2; 1%) (Table 2). The odds ratio for patient delay in seeking care was highest among patients with TBI (OR 22.4), and concussion (OR 4.1). There were no statistically significant differences in reason for delay in diagnosis by age, sex, race/ethnicity, or trauma alert level.

**Table 2:** Reasons for Delay in Diagnosis of Intracranial Trauma

Reason	N	%
Patient did not seek care	111	79
Diagnosis not made at initial ED visit and patient discharged	9	6
Diagnosis not made at other outpatient facility	4	3
Diagnosis not made initially during inpatient hospitalization	16	11
Alcohol intoxication	4	3
Other injuries	9	6
Mental health issue	2	1

## Discussion

In this study, the most common types of delayed diagnoses included subdural hemorrhage and intraparenchymal hemorrhage, subarachnoid hemorrhage, and TBI. The Centers for Disease Control and Prevention (CDC) defines TBI as "a disruption in the normal function of the brain that can be caused by a bump, blow, or jolt to the head, or penetrating head injury" [11]. TBIs can be classified into two different types: open-head injury and closed-head injury. The majority of the patients in this study had a nonpenetrating or closed-head injury. Taking this into consideration, it must be known that nonpenetrating TBIs are much harder to identify due to the obscurity combined with the ability of the patient to detect intracranial trauma symptoms. Symptoms consistent with a more obvious TBI likely to prompt medical care include but are not limited to posttraumatic amnesia, disorientation, confusion, and seizure without or without a history of seizure disorder. TBI symptoms that may appear as less severe to the patient and delay presentation to a medical facility include temporary and/or quickly resolved visual field deficits, nausea, vomiting, and generalized fatigue and weakness. Despite severity, these symptoms typically arise immediately after the traumatic event and persist with a variable recovery timeframe, depending on the patient. 

Delays in seeking medical care may be multifactorial. Previous studies have demonstrated that delay may occur due to resource availability (including transportation, finances or insurance, homelessness), subjective interpretation of symptoms (minimal versus severe), fear of medical care, fear of infection, fear of overcrowded hospitals, or financial concerns [12-17]. The National Healthcare for the Homeless Council in 2019 stated that homeless patients have higher rates of illness and die 12 years earlier than the general population in the United States [18]. Homelessness is also associated with infection and illness exposure, lack of protection from the environment, and decreased adherence to prescription medication [19]. In addition, psychiatric illness and substance abuse are more likely to occur in those who are homeless or lack resources. 

This study found that among inpatients with a delayed diagnosis of intracranial trauma, alcohol intoxication, and distracting injuries were common. There was no association with delayed diagnosis and age, sex, race/ethnicity, or trauma alert level. These data contradict a published study that identified trauma activation and plain film imaging associated with delayed diagnosis of trauma [20].

Delay in diagnosis may occur from distraction or cognitive bias. Severe injuries may be a distraction from recognition of intracranial trauma. Cognitive bias may also result in delay of diagnosis, including confirmation bias, and affect heuristic, premature closure, or outcomes bias [21-23]. Anchoring bias may account for delayed diagnosis in the presence of other injuries [24-27]. Metacognition is an approach to overcoming cognitive errors and should include a reflective approach that involves stepping back from the immediate problem to examine and reflect on the thinking process [28].

This study has several limitations. As a retrospective study, the data are dependent on accurate data entry. These data are from a single institution and may not be generalizable to other locations.

## Conclusions

Among patients with delayed diagnosis of intracranial trauma, the majority of delays in diagnosis were due to patient delay in seeking care. Other reasons for delay in diagnosis included the presence of other injuries, mental health issues, and alcohol intoxication. Future directions may include improved public education regarding trauma, the importance of seeking timely medical care, and provider education regarding cognitive bias.
